# Automatic Abstraction of Computed Tomography Imaging Indication Using Natural Language Processing for Evaluation of Surveillance Patterns in Long-Term Lung Cancer Survivors

**DOI:** 10.1200/CCI-24-00279

**Published:** 2025-07-23

**Authors:** Aparajita Khan, Eunji Choi, Chloe Su, Anna Graber-Naidich, Solomon Henry, Mina L. Satoyoshi, Archana Bhat, Allison W. Kurian, Su-Ying Liang, Joel Neal, Michael Gould, Ann Leung, Heather A. Wakelee, Leah M. Backhus, Curtis Langlotz, Julie Wu, Summer S. Han

**Affiliations:** ^1^ Department of Computer Science and Engineering, Indian Institute of Technology (BHU) Varanasi, Varanasi, India; ^2^ Quantitative Sciences Unit, Department of Medicine, Stanford University School of Medicine, Stanford, CA; ^3^ Population Health Sciences, Weill Cornell Medical College, New York, NY; ^4^ Department of Epidemiology and Population Health, Stanford University, Stanford, CA; ^5^ Department of Biomedical Data Science, Stanford University, Stanford, CA; ^6^ Research Informatics Center, Stanford University, Stanford, CA; ^7^ Department of Medicine, Stanford University School of Medicine, Stanford, CA; ^8^ Palo Alto Medical Foundation Research Institute, Sutter Health, Palo Alto, CA; ^9^ Division of Oncology, Department of Medicine, Stanford University School of Medicine, Stanford, CA; ^10^ Department of Health Systems Science, Kaiser Permanente Bernard J. Tyson School of Medicine, Pasadena, CA; ^11^ Department of Radiology, Stanford University School of Medicine, Stanford, CA; ^12^ Stanford Cancer Institute, Stanford University School of Medicine, Stanford, CA; ^13^ Division of Thoracic Surgery, Department of Cardiothoracic Surgery, Stanford University School of Medicine, Stanford, CA; ^14^ Palo Alto VA, Palo Alto, CA; ^15^ Department of Neurosurgery, Stanford University School of Medicine, Stanford, CA

## Abstract

**PURPOSE:**

Despite its routine use to monitor patients with lung cancer (LC), real-world evaluations of the impact of computed tomography (CT) surveillance on overall survival (OS) have been inconsistent. A major confounder is the absence of imaging indications because patients undergo CT scans for purposes beyond surveillance, like symptom evaluations (eg, cough) linked to poor survival. We propose a novel natural language processing model to predict CT imaging indications (surveillance *v* others).

**METHODS:**

We used electronic health records of 585 long-term LC survivors (≥5 years) at Stanford, followed for up to 22 years. Their 3,362 post–5-year CT reports (including 1,672 manually annotated) were used for modeling by integrating structured variables (eg, CT intervals) with key-phrase analysis of radiology reports. Naïve analysis compared OS in patients with CT for any indications (including symptoms) versus those without post–5-year CT, as in previous studies. Using model-predicted indications, we conducted exploratory analyses to compare OS between those with post–5-year surveillance CT and those without.

**RESULTS:**

The model showed high discrimination (AUC, 0.86), with key predictors including a longer interval (≥6-month) from the previous CT (odds ratios [OR], 5.50; *P* < .001) and surveillance-related key phrases (OR, 1.37; *P* = .03). Propensity-adjusted survival analysis indicated better OS for patients with any post–5-year surveillance CT versus those without (adjusted hazard ratio, 0.60; *P* = .016). By contrast, no significant survival difference was found (*P* = .53) between patients with any CT versus those without post–5-year CT.

**CONCLUSION:**

Our model abstracted CT indications from real-world data with high discrimination. Exploratory analyses revealed the obscured imaging-OS association when considering indications, highlighting the model's potential for future real-world studies.

## INTRODUCTION

Advances in early lung cancer (LC) detection and treatment have significantly improved 5-year LC survival in the United States,^1,2^ with an estimated 654,620 LC survivors as of 2022.^1^ These LC survivors, however, face a high risk of developing second primary LC (SPLC), with an approximate 10-year risk of 8.36% following a 5-year survival,^3-6^ with subsequently increased LC mortality.^6-8^ However, guidelines on long-term surveillance strategies to detect second malignancies remain elusive.^9-11^ Although guidelines from the National Comprehensive Cancer Network^12^ and ASCO^13^ recommend annual computed tomography (CT) scans every 6 months for 2 years after curative treatment and then annually, these recommendations are primarily based on expert consensus^13-15^ without robust evidence. For long-term survivors beyond 5 years of survival, the optimal cadence for post-treatment surveillance is even less certain.^9,16^

**CONTEXT**

**Key Objective**
Can integrating structured and unstructured electronic health records (EHRs) enable the abstraction of computed tomography (CT) imaging indications and assess the impact of CT surveillance on overall survival (OS) among long-term lung cancer (LC) survivors?
**Knowledge Generated**
Among 585 patients with LC who survived ≥5 years from initial diagnosis, an integrative model combining structured EHRs and 3,362 CT reports predicted imaging indications with high discrimination (AUC, 0.86). Propensity-adjusted analysis revealed significantly improved OS for patients with post–5-year surveillance CT compared with those without (adjusted hazard ratio, 0.60), whereas naïve analyses that did not account for imaging indications showed no significant difference between patients with any CT and those without.
**Relevance *(J.L. Warner)***
The presented work demonstrates how to automatically evaluate CT indications, which is very valuable in identifying the impact of LC screening versus other CTs.*
*Relevance section written by *JCO CCI* Editor-in-Chief Jeremy L. Warner, MD, MS, FAMIA, FASCO.

Although prospective randomized controlled trials are considered the gold standard for generating high-quality evidence, their practicality and statistical power could be limited in assessing the efficacy of SPLC surveillance.^17,18^ Not only would the cost be prohibitory because of a long follow-up period, but ethical issues also surround randomly assigning patients with diagnosed LC to surveillance versus no surveillance. Consequently, real-world evidence studies become pivotal for evaluating the potential impact of SPLC surveillance among long-term survivors. However, a significant challenge in conducting these studies is rooted in the lack of readily available data indicating the purpose of the imaging examinations received. Currently, it is impossible to differentiate between imaging scans performed for surveillance of new malignancies versus those performed for other reasons (eg, symptoms or metastasis treatment) without a laborious and time-consuming manual review of patients' medical records. Thus, although some retrospective studies have attempted to establish evidence of surveillance benefits, the findings have often conflicted (survival advantage^10,19^ *v* no significant benefit^11,20^) as their ability to assess true benefit of surveillance is restricted by their lack of ability to account for imaging indications.

To address the critical gap in distinguishing CT scans performed for SPLC surveillance from those conducted for other purposes, such as symptom evaluation or metastasis monitoring, in this study, we aimed to develop a novel natural language processing (NLP)–based model to predict CT imaging indications (surveillance *v* others). This can eliminate the future need for manual review of radiology reports and enable robust evaluations of long-term surveillance strategies for SPLC. To this end, we used a hybrid approach leveraging both granular clinical details from 1,672 manually annotated chest CT reports and structured electronic health record (EHR) data (eg, ordering provider specialty and diagnosis codes). We further explored the model's clinical utility by evaluating the association between CT surveillance and survival among long-term survivors (≥5 years) considering the predicted imaging indications.

## METHODS

### Study Cohort

**FIG 1.**
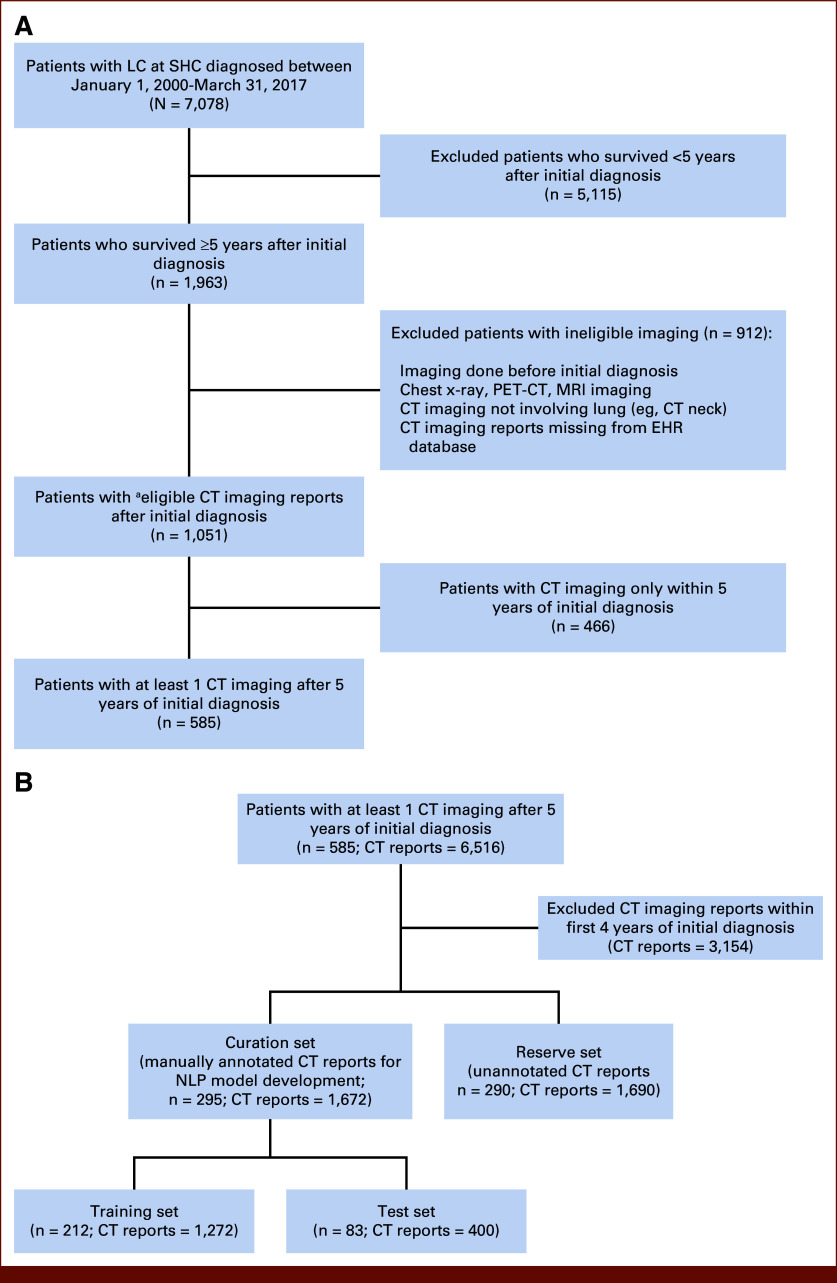
Cohort derivation and partitioning of CT reports for NLP-based modeling for imaging indication. (A) Cohort selection: Selection of the study cohort from patients with LC at SHC for CT indication modeling using NLP. (B) Radiology notes used for NLP model training and validation: Partitioning of CT radiology reports of patients with post–5-year CT into a curation set of manually annotated reports for NLP model development and a reserve set for predicting CT indications on unannotated reports. ^a^Noneligible imaging was defined as imaging that was performed before the initial diagnosis or that involved chest x-rays, PET-CTs, MRI scans, imaging that did not involve the lung (eg, CT neck), and imaging whose CT reports were absent from the EHR database. CT, computed tomography; EHR, electronic health record; LC, lung cancer; MRI, magnetic resonance imaging; NLP, natural language processing; PET, positron emission tomography; SHC, Stanford Health Care.

Of the 7,078 patients diagnosed with LC at Stanford Health Care (SHC) between January 1, 2000, and March 31, 2017, we identified 1,963 patients who survived at least 5 years after the initial LC diagnosis. Their LC diagnoses were confirmed using the Stanford's local tumor registry. The study cohort comprised 585 patients who had at least one chest CT scan completed after 5 years of survival, with total of 3,362 CT reports (Fig 1A), which was used for modeling imaging indications. Patients were followed until death or censored at their last SHC visit. Stanford Institutional Review Board approved the study with informed consent waived because of minimal risk.

### Study Outcomes

The primary study outcome was the AUC metric^21^ to evaluate the predictive performance of the NLP-based model for classifying each CT imaging indication as surveillance versus others. Model performance was further assessed using calibration, sensitivity, and specificity (Data Supplement, Method S1). The secondary outcome was overall survival (OS) from the 5-year survival of the initial diagnosis, which was associated with the receipt of at least one post–5-year surveillance CT scan.

### Model Development and Validation for Abstracting CT Imaging Indication

To predict the indication of each of the 3,362 chest CT scans from 585 patients using NLP modeling, we randomly split the 585 patients into a curation set (1,672 CT reports from 295 patients) and a reserve set (1,690 CT reports from 290 patients) (Fig 1B and Table 1). To construct the ground truth label for model development, three independent annotators, supervised by a medical oncologist, manually reviewed the 1,672 reports in the curation set, labeling each as surveillance or other indications (Data Supplement, Method S2 and Fig S1). Of the curation set, approximately 70% (1,272 CT reports) were used as the NLP model training set and the remaining (400 CT reports) were used as the test set to evaluate the performance of the models (Fig 1B).

**TABLE 1.** Baseline Characteristics and Follow-Up Time for Patients in the Study Cohort (used for NLP modeling)

Characteristic	Total	Curation Set^a^ (manually annotated)	Reserve Set
No. of patients (%)	585	295	290
No. of CT reports	3,362	1,672	1,690
Age			
Median (IQR)	65.5 (57.9-71.7)	65.7 (58.7-71.8)	65 (56.3-71.7)
Sex, No. (%)			
Male	223 (38.1)	120 (40.7)	103 (35.5)
Female	362 (61.9)	175 (59.3)	187 (64.5)
Race/ethnicity, No. (%)			
White	306 (52.3)	147 (49.8)	159 (54.8)
Black	13 (2.2)	5 (1.7)	8 (2.8)
Hispanic	26 (4.4)	13 (4.4)	13 (4.5)
Asian	178 (30.4)	98 (33.2)	80 (27.6)
Other	32 (5.5)	15 (5.1)	17 (5.9)
Missing	30 (5.1)	17 (5.8)	13 (4.5)
Stage, No. (%)			
I	295 (50.4)	139 (47.1)	156 (53.8)
II	45 (7.7)	20 (6.8)	25 (8.6)
IIIA	56 (9.6)	28 (9.5)	28 (9.7)
IIIB/C	44 (7.5)	26 (8.8)	18 (6.2)
IV	107 (18.3)	58 (19.7)	49 (16.9)
Missing	38 (6.5)	24 (8.1)	14 (4.8)
Histology, No. (%)			
Adenocarcinoma	432 (73.8)	216 (73.2)	216 (74.5)
NSCLC	28 (4.8)	16 (5.4)	12 (4.1)
Large cell	4 (0.7)	3 (1)	1 (0.3)
Small cell	6 (1)	4 (1.4)	2 (0.7)
Squamous cell	59 (10.1)	27 (9.2)	32 (11)
Other	56 (9.6)	29 (9.8)	27 (9.3)
Smoking status, No. (%)			
Active	22 (3.8)	11 (3.7)	11 (3.8)
Former	284 (48.5)	142 (48.1)	142 (49)
Never	269 (46)	140 (47.5)	129 (44.5)
Missing	10 (1.7)	2 (0.7)	8 (2.8)
Surgery, No. (%)			
Yes	392 (67)	194 (65.8)	198 (68.3)
No	192 (32.8)	101 (34.2)	91 (31.4)
Missing	1 (0.2)	0 (0)	1 (0.3)
Chemotherapy, No. (%)			
Yes	234 (40)	122 (41.4)	112 (38.6)
No	350 (59.8)	173 (58.6)	177 (61)
Missing	1 (0.2)	0 (0)	1 (0.3)
Radiation therapy, No. (%)			
Yes	142 (24.3)	69 (23.4)	73 (25.2)
No	442 (75.6)	226 (76.6)	216 (74.5)
Missing	1 (0.2)	0 (0)	1 (0.3)
Follow-up time			
Median (IQR)	8.3 (6.7-10.7)	8.2 (6.6-10.3)	8.4 (6.8-11.1)

Abbreviations: CT, computed tomography; NLP, natural language processing; NSCLC, non–small cell lung cancer.

a The curation set consists of patients whose CT reports were manually reviewed by annotators and labeled as either surveillance or other indications for model development, whereas the reserve set consists of patients with unannotated CT reports whose indications are not manually labeled but are instead determined on the basis of the predictions made by the model.

We developed three different models to predict CT imaging indication using 10-fold cross-validation in the training set: (1) the model solely based on the NLP-derived variables abstracted from radiology reports (see NLP Algorithms and Feature Extraction section, Data Supplement, Methods S3, S4, and Table S1), (2) the model solely based on the structured EHR variables (see Structured Variables for Predicting CT Imaging Indication section, Data Supplement, Method S5 and Table S2), and (3) the hybrid model that uses both the structured and NLP-derived variables. Variable selection involved testing the univariate association of individual features (listed in Data Supplement, Table S3), followed by various selection strategies guided by model performance as described in the Data Supplement (Method S6). After model training, we compared the models' performance across metrics (Data Supplement, Methods S1 and S7) using the test set (400 reports).

### NLP Algorithms and Feature Extraction

The NLP features for predicting CT imaging indications were abstracted from free-text CT reports, the structure of which is described in the Data Supplement (Method S3). We developed a six-step NLP pipeline (Data Supplement, Method S4 and Fig S2), including (1) segmentation, (2) tokenization,^22^ (3) parts of speech tagging,^23^ (4) key-phrase identification,^24,25^ (5) clustering of key phrases by oncology concepts (Data Supplement, Fig S3, shows the clusters contents), and (6) frequency-based feature extraction. A total of 11 NLP-derived variables were extracted for model development (Data Supplement, Table S1).

### Structured Variables for Predicting CT Imaging Indication

We considered seven variables obtained from structured EHR data for predicting imaging indications (Data Supplement, Method S5 and Table S2), including ordering provider specialty (eg, oncology, emergency medicine), the numbers of symptom-related International Classification of Diseases (ICD) codes (eg, cough, chest pain), and lung disease-related ICD diagnosis codes (eg, pneumonia, hemoptysis) within the past 6 months and the time since previous CT scans (Data Supplement, Tables S4-S6).

### Statistical Analysis

#### Analysis of Temporal Patterns of CT Surveillance

Measuring adherence to guideline-recommended imaging-based surveillance is important for assessing the quality of care and identifying areas for improvement. However, actual adherence to the recommended annual surveillance CT interval for long-term LC survivors is unknown because of the lack of indication for CT imaging in previous studies, making it challenging to differentiate between routine surveillance and diagnostic scans.

To this end, we applied the proposed hybrid model to predict the imaging indication for each of the 3,362 CT radiology reports both in the curation set and the reserve set (n = 585; Fig 1B). Using model-predicted CT imaging indications, we analyzed the temporal patterns of CT surveillance beyond 5 years after diagnosis (Data Supplement, Method S8). We assessed adherence to annual CT surveillance recommendations using a sliding window technique (Data Supplement, Method S8 and Fig S4A) to differentiate between surveillance CT scans adhering to regular, *annual* surveillance versus those acquired irregularly (Data Supplement, Fig S5).

#### Survival Analysis

To evaluate the clinical utility of the proposed model for CT imaging indications, we conducted an exploratory analysis examining the association between OS and the receipt of surveillance CT after 5-year survival among long-term LC survivors.

Before this analysis, we performed a naïve counterpart analysis to assess the association between OS and the exposure comparison group defined as those who received *any* CT scans (regardless of imaging indications, including both surveillance and other reasons) versus those who received no CT beyond 5-year survival, using a commonly employed exposure definition (any CT *v* no CT) found in the literature.^19,20^ For the control group without any CT, we used an additional cohort of 128 patients who (1) survived at least 5 years, (2) continued to receive active follow-up care at SHC as indicated by interactions such as out/in-patient, office, laboratory, or emergency visits, but (3) had no CT scans completed after 5-year survival (Data Supplement, Table S7). Patients in this additional cohort were selected to have a matching distribution of follow-up duration with those in our cohort who received post–5-year CT (Data Supplement, Method S9 and Fig S6).

To evaluate the association between OS and receipt of surveillance CT, we applied multivariate Cox regression, in which the primary exposure group was defined as those who received at least one surveillance CT (predicted using the model) versus those who received no CT after 5-year survival from the initial diagnosis (n = 128). The model was adjusted for potential confounders, including sex, race/ethnicity, cancer stage, and histology, and used the inverse probability treatment weighting (IPTW)^26^ to generate a pseudo-population where exposure allocation is independent of confounders (see Data Supplement, Method S10).

#### Generalizability Analysis Using SEER

Considering that the patient characteristics at SHC may differ from those in the general population (eg, a higher proportion of Asian patients or having better survival), we conducted a sensitivity analysis to evaluate the external validity and generalizability of our study findings using the weights obtained by analyzing additional 5,700 5-year LC survivors from the national SEER cancer registry^27^ (Data Supplement, Method S11 and Table S8). We applied a standardization method to reweight the SHC patient cohort to be standardized to the characteristics of patients with LC in SEER to improve the generalizability of the association findings obtained from SHC.

#### Sensitivity Analysis on Early-Stage Patients

Additionally, we performed two sensitivity analyses, one focusing on patients with early-stage (I-IIIA) LC (n = 396) and another on patients with stage I-II LC who underwent surgery (n = 296), to evaluate the robustness of our results and reduce potential confounding from ongoing metastatic care among advanced-stage patients.

All statistical analyses were performed using R version 3.4 (R Foundation for Statistical Computing, Vienna, Austria).

## RESULTS

### Patient Characteristics

The 585 patients in the SHC cohort—who survived at least 5 years from initial diagnosis—had a median diagnosis age of 65.5 years (IQR, 57.9-71.7) and were primarily female (61.9%), White (52.3%), and former smokers around the 5-year survival mark (48.5%). Of these survivors, 396 (67.7%) had early-stage (I-IIIA) LC, with adenocarcinoma as the most common histology (73.8%) (Fig 1A and Table 1).

### NLP-Based Hybrid Model Performance

**FIG 2.**
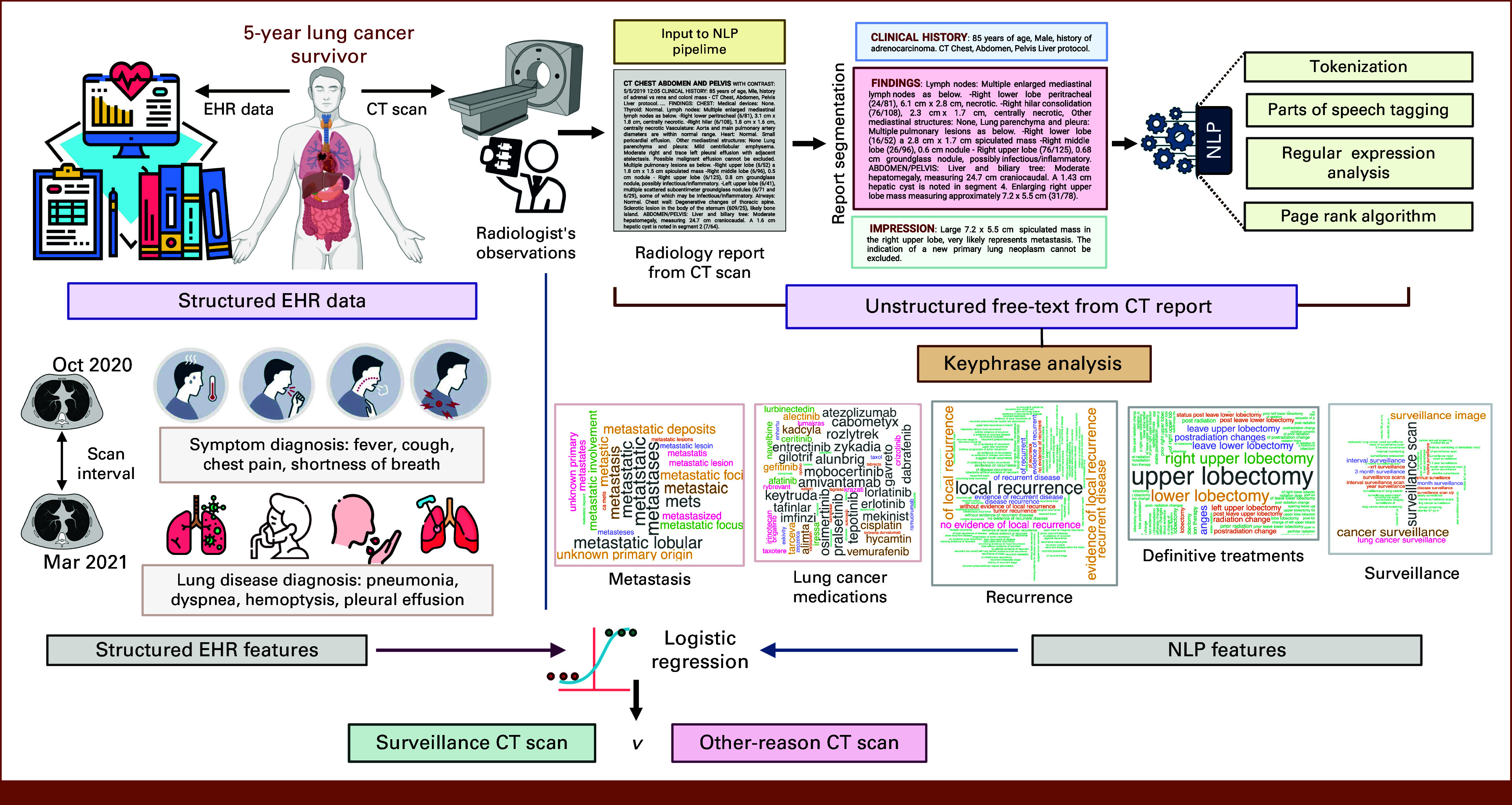
Schematic diagram of proposed hybrid model. The development of the proposed hybrid NLP-based model for predicting CT imaging indications involved an intricate process of integrating and harmonizing data from structured EHRs and unstructured CT reports. Left half: The model extracts various features from structured EHRs (such as CT scan intervals, patient symptoms, and lung disease diagnoses, primarily using ICD9/10 diagnosis codes and CPT procedure codes; see Data Supplement, Method S5). Right half: Unstructured free-text CT radiology reports are processed using a six-step NLP pipeline (outlined in the Data Supplement, Method S4) to extract the occurrence frequency of key phrases related to various aspects of lung cancer, like surveillance, recurrence, metastasis, treatments, and medications, from the CT report. After extracting these two distinct sets of features, the model employs multivariate logistic regression to combine these features to predict/classify the indication of each CT imaging into either surveillance or other reasons (eg, symptoms or metastasis treatment). CPT, current procedural terminology; CT, computed tomography; EHR, electronic health record; ICD, International Classification of Diseases; NLP, natural language processing.

**FIG 3.**
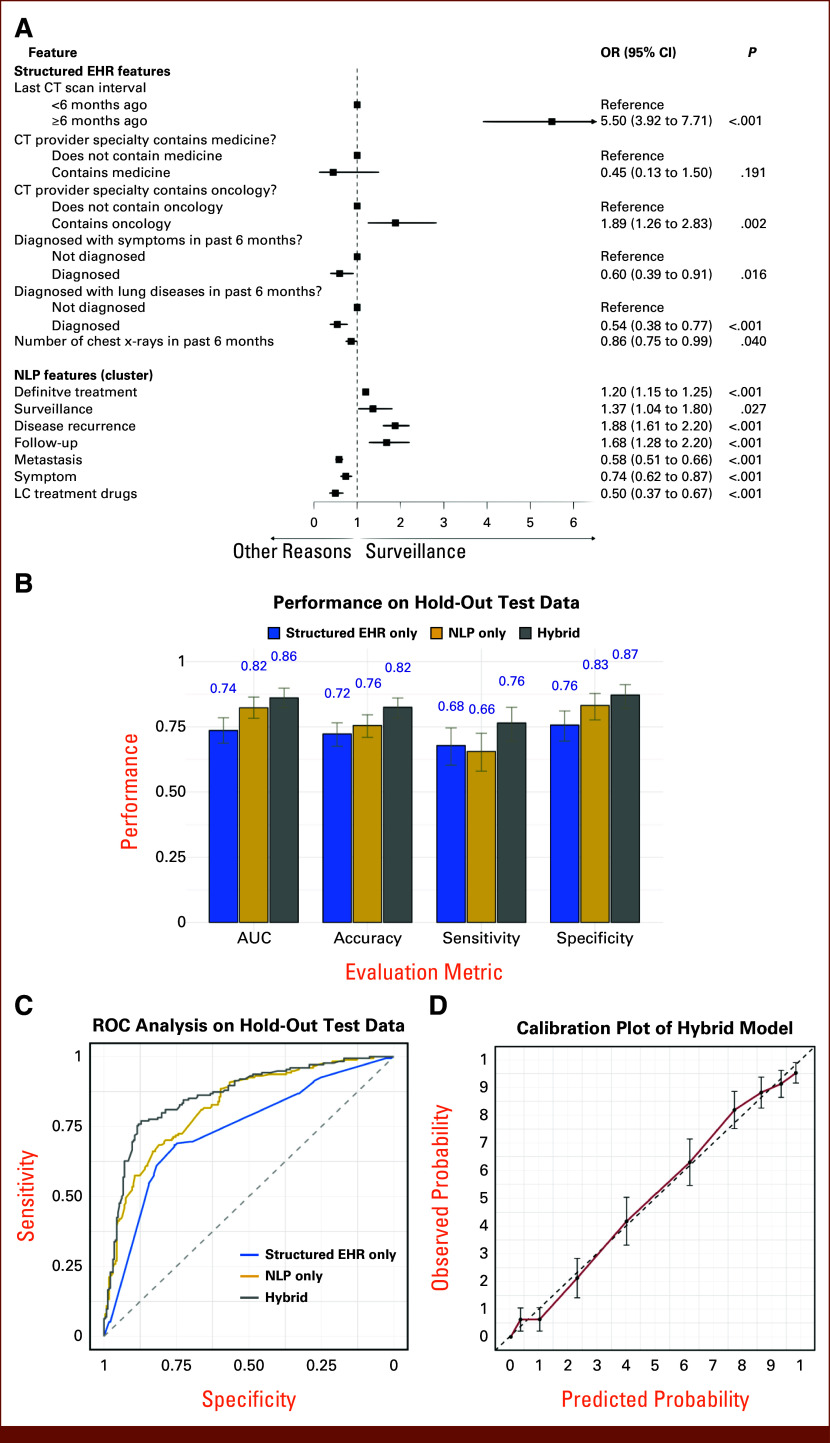
Analysis of model performance and association with CT imaging indications. (A) The features included in the proposed hybrid NLP-based model, with the corresponding forest plot illustrating the association between each feature (both structured EHR and NLP features) and the probability that the given CT scan was performed because of surveillance (*v* others); this was estimated through multivariate logistic regression. Square symbols represent OR estimates, whereas error bars denote the 95% CIs. (B) The comparative performance of the proposed hybrid model against the models using solely structured EHRs or NLP feature subsets, evaluated on a hold-out test data set. Metrics assessed include the AUC, classification accuracy, sensitivity, and specificity (Data Supplement, Method S1). (C) ROCs for the proposed hybrid model, structured EHR-only model, and NLP-only model, displaying their performance on test data. (D) Calibration plot for the proposed model, illustrating the agreement between model-predicted probabilities and observed probabilities in the data. The 45-degree dashed diagonal line represents perfect calibration, with the plotted line in red indicating the actual model performance. CT, computed tomography; EHR, electronic health record; LC, lung cancer; NLP, natural language processing; OR, odds ratios; ROC, receiver operating curve.

Our hybrid NLP-based model for predicting CT imaging indications (Fig 2), trained on 1,272 CT reports, identified several variables positively associated with a higher likelihood of surveillance indication (*v* others such as symptoms or metastasis treatment). These included a longer interval (≥6 *v* <6 months) from prior CT scans (odds ratios [OR], 5.5 [95% CI, 3.91 to 7.71]; *P* < .001) and occurrences of key phrases related to surveillance (OR, 1.37 [95% CI, 1.04 to 1.80]; *P* = .027) and follow-up (OR, 1.68 [95% CI, 1.28 to 2.2]; *P* < .001) (Fig 3A). On the hold-out test set, the proposed model exhibited good discrimination (Figs 3B and 3C; AUC, 0.86 [95% CI, 0.82 to 0.90]), outperforming the models solely based on NLP features (AUC, 0.82 [95% CI, 0.78 to 0.86]; Data Supplement, Fig S7A) and structured variables (AUC, 0.74 [95% CI, 0.68 to 0.78]; Data Supplement, Fig S7B), with good model calibration (Fig 3D and Data Supplement, Fig S8). The validated model is accessible as free software, the DistinCT R-package.^28^

### Temporal CT Surveillance Patterns and Adherence to Annual Surveillance Guidelines

**FIG 4.**
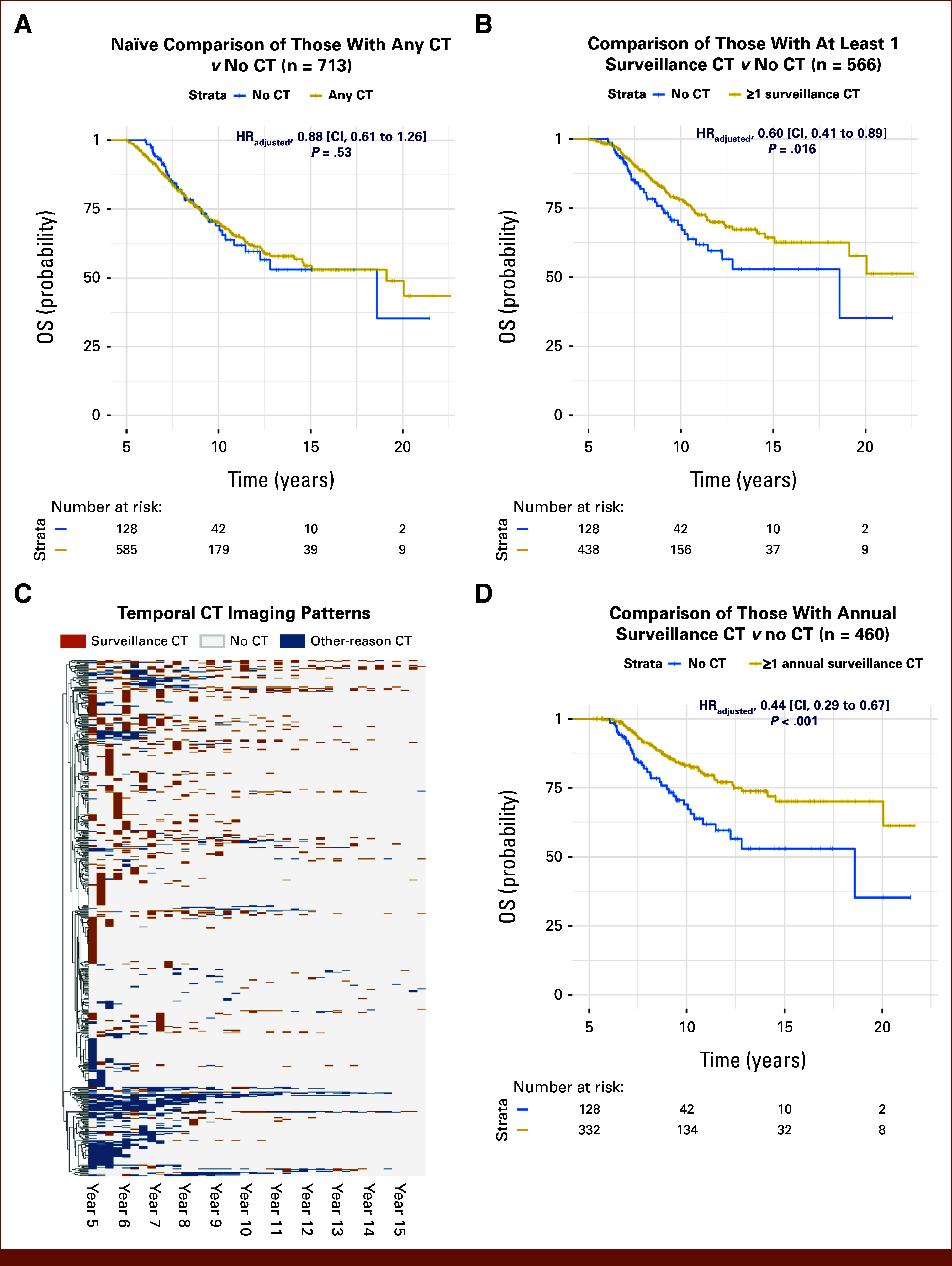
Evaluating associations between OS and CT surveillance using predicted imaging indications and characterization of temporal patterns in CT surveillance. (A) Naïve survival analysis for OS and any CT scans without imaging indication shows the Kaplan-Meier plot comparing OS between those who received *any* CT scans (either surveillance or others; n = 585) beyond 5-year survival—thus, not considering imaging indications—and those who did not receive any CT scans (n = 128, in black) beyond 5-year survival whose follow-up duration was matched; the hazard ratio for the association between OS and CT scans was derived using multivariate Cox regression, adjusting for sex, race/ethnicity, initial cancer stage, and histology at diagnosis, with additional correction for selection bias through inverse probability treatment weighting to balance patient characteristics. (B) Survival analysis for OS and CT surveillance using model-predicted imaging indications: Kaplan-Meier plot for analogous analysis conducted in (A) but comparing OS stratified by (1) the patients who received at least one CT classified as surveillance based on the proposed hybrid model (n = 438, in orange) versus (2) those who did not receive any CT scans (n = 128, in black) beyond 5-year survival whose follow-up duration was matched; (C) visualization of temporal surveillance patterns beyond 5 years from the initial diagnosis: Heat map with rows representing patients, columns depicting quarterly time points for 10 years from the 5-year survival, and cell colors indicating model-predicted CT indications (orange: surveillance CT, blue: other reason CT, white: no CT). Hierarchical clustering of patients (rows) based on temporal CT indication profiles revealed two patient groups: one primarily composed of patients with other-reason CT scans (bottom half of heat map), while the other comprised patients with a mix of surveillance and other-reason scans (upper half of heat map). (D) Survival analysis for OS and *annual* CT surveillance based on temporal analysis shows the analogous analysis conducted in (A) but comparing OS between (1) those who received at least one *annual* surveillance CT scan (n = 332, in orange) beyond 5-year survival—identified through a moving window approach (see Data Supplement, Method S8) considering imaging indications—and (2) those who did not receive any CT scans (n = 128, in black) beyond 5-year survival. CT, computed tomography; HR_adjusted_, adjusted hazard ratio was obtained using multivariate Cox regression, adjusting for sex, race/ethnicity, and initial cancer histology at diagnosis, with additional correction for selection bias through inverse probability treatment weighting to balance patient characteristics; OS, overall survival.

We used the proposed model to characterize the temporal surveillance patterns in LC survivors beyond 5-year survival at SHC. We applied the model to predict the imaging indication for each of the 3,362 CT reports both in the curation and reserve sets (n = 585). We observed the highest proportion of patients who received at least one surveillance CT around the 5-year survival mark, which decreased over time (Data Supplement, Fig S9). Clustering analysis based on the predicted temporal surveillance patterns identified several distinct groups of survivors (Fig 4C), including those with frequent surveillance CT scans (in orange) and those mostly with CT scans only for other reasons, for example, symptoms (in blue).

Following the temporal CT surveillance analysis, we evaluated the patterns of adherence to *annual* surveillance guidelines (Data Supplement, Fig S4B) as recommended by current clinical guidelines (eg, National Comprehensive Cancer Network). We found that 56.8% (n = 332) of patients in the SHC cohort received at least one regular, annual surveillance CT beyond 5-year survival.

### Exploratory Analysis to Evaluate Association Between OS and Long-Term CT Surveillance

To evaluate the clinical utility of the proposed model, we conducted an exploratory analysis to estimate the association between CT surveillance and OS among long-term LC survivors, considering the predicted imaging indications. According to the predicted indications, among 585 LC survivors, 75% (n = 438) received at least one surveillance CT beyond 5-year survival, whereas 147 (25%) received CT only for other nonsurveillance reasons (Data Supplement, Fig S5 and Table S9). Patients with higher comorbidities (Charlson Comorbidity Index,^29^ *P* < .001) and those diagnosed with lung conditions such as pneumonia, pleural effusion, or dyspnea (see Data Supplement, Table S6) within 1 year before 5-year survival were more likely to undergo only nonsurveillance CTs rather than surveillance CTs (Data Supplement, Table S9).

In a naïve analysis that did not consider CT imaging indications, no significant difference in OS was observed between those who received CT with *any* indications (surveillance or others; n = 585) versus the matched cohort of patients without any post–5-year CT (n = 128; adjusted hazard ratio [HR_adjusted_], 0.88 [95% CI, 0.61 to 1.26]; *P* = .53) (Fig 4A), a typically used comparison grouping method in the literature. When the predicted imaging indication was taken into account, the patients who received ≥1 surveillance CT beyond 5-year survival (n = 438) showed better OS versus those in the matched cohort without any post–5-year CT (n = 128; HR_adjusted_, 0.60 [95% CI, 0.41 to 0.89]; *P* = .016) (Fig 4B).

Sensitivity analysis to evaluate the external validity of the study findings using the propensity weights obtained by analyzing an external cohort of 5,700 LC survivors from the national SEER registries showed consistent results (Data Supplement, Fig S10 and Table S8). A subanalysis on patients with early-stage (I-IIIA) LC (n = 396) demonstrated consistent findings for both the primary and naïve analyses (Data Supplement, Fig S11 and Table S10). Additionally, a more focused subanalysis of surgically treated stage I-II patients (n = 296) revealed a consistent trend toward improved OS in those receiving ≥1 surveillance CT beyond 5-year survival compared with matched patients without post–5-year CT (Data Supplement, Table S11 and Fig S12) further supporting the potential value of surveillance imaging in this population. Additional analysis that compared OS between those who received ≥1 guideline-concordant *annual* surveillance CT versus those without any post–5-year CT showed similar results as our primary analysis (Figs 4B and 4D, Data Supplement, Table S12).

## DISCUSSION

In this study, we proposed a novel NLP-based method for automated abstractions of CT imaging indications by integrating structured and unstructured EHR data. This method demonstrated high discrimination, accuracy, and calibration in predicting CT imaging indications. We further explored the model's clinical utility by estimating the association between OS and the receipt of CT surveillance predicted using the proposed model among LC survivors. This analysis showed a potential association between better OS and the receipt of at least one surveillance CT beyond 5-year survival among the survivors.

Although several previous studies proposed NLP algorithms for radiology reports,^30-41^ most focused on interpreting radiologic findings, such as identifying concerning nodules/lesions,^30,31^ actionable findings,^32-35^ and follow-up recommendations.^30-32,37-40^ However, few studies aimed to identify the scan's intended purpose, which is critical in evaluating the potential benefits of imaging surveillance in patients with cancer. Our study designed features indicative of the imaging purpose rather than its findings, identifying interpretable key phrases associated with surveillance (eg, follow-up) or nonsurveillance (eg, symptoms) purposes. This sets the model apart from black-box approaches of the recent large language models and neural network models,^42,43^ also helping surpass previous semiautomated NLP methods to classify CT imaging indications that relied on a combination of manual abstractions and automated key-phrase identification.^44^

To the best of our knowledge, this study is among the first to develop an integrative NLP-based model to autonomously abstract CT imaging indications in patients with cancer, incorporating comprehensive structured and unstructured EHR data. To improve model performance, we used various NLP methods, including parts of speech tagging and graph-based key-phrase identification. The robustness of the study's manual annotations, conducted by three annotators with evaluated concordance, underscores the reliability of the data used for model development. Using these annotated data, we developed and compared multiple models, assessing their performance under various metrics to evaluate the proposed model's robustness. Additionally, the utility of the proposed model was explored by characterizing temporal CT surveillance patterns in long-term survivors. To facilitate broader usage, the proposed CT indication model is implemented and available as the *DistinCT* R-package.^28^

This study has several limitations. First, being based on single-institution data could affect the generalizability of the study findings, with a limited sample size and potential missing data for long-term follow-up. However, to improve generalizability, we used the national SEER registry data and applied a rigorous standardization method to reweight the SHC cohort to match SEER patient characteristics, which yielded consistent findings. As patients may relocate or change health care providers, selection bias may exist among those who did not receive any CT beyond 5 years from the initial diagnosis. To avoid potential selection bias because of missingness for follow-up, we used a causal inference method on the basis of IPTW and matched follow-up durations across comparison groups. We also observed that a greater proportion of scans performed for nonsurveillance purposes were performed with contrast. Additionally, patients with recent acute health care utilization, such as emergency or inpatient visits within 1 month prior, were more likely to undergo CTs for nonsurveillance purposes. Incorporating factors like contrast use and acute health care utilization into the NLP model could enhance its accuracy in distinguishing surveillance CTs from other imaging types. Finally, we were unable to investigate the impact of CT surveillance on the early detection of SPLC because of the absence of specific structured data for its diagnoses in the EHRs, which would be a meaningful next step.

In conclusion, this study showcases an innovative tool for extracting imaging indications by integrating structured and unstructured EHRs. Our result demonstrates that CT indications can be abstracted with high discrimination from real-world data, facilitating analysis for evaluating the potential survival impact of CT surveillance among long-term LC survivors.
